# The likely role of proteolytic enzymes in unwanted differentiation of stem cells in culture

**DOI:** 10.4155/fso.15.26

**Published:** 2015-11-01

**Authors:** Vanessa Penna, Monica VN Lipay, Monica T Duailibi, Silvio E Duailibi

**Affiliations:** 1 Graduate Translational Surgery Program, Paulista School of Medicine, Federal University of Sao Paulo (EPM/UNIFESP), SP, Brazil; 2 CTCMol, Center of Cellular & Molecular Therapy, São Paulo, SP, Brazil; 3 Department of Morphology & Basic Pathology, Jundiaí Faculty of Medicine, São Paulo, SP, Brazil; 4 Medicine Department, Endocrinology Division, EPM/UNIFESP, Sao Paulo, SP, Brazil; 5 Surgery Department, Translational Surgery Division, EPM/UNIFESP, Sao Paulo, SP, Brazil; 6 Department of Implantology, University Santo Amaro – UNISA, São Paulo, SP, Brazil

**Keywords:** cell communication, cell culture, extracellular matrix, mechanotransduction, proteolytic enzymes, regenerative medicine, stem cells, surface proteins, tissue engineering

## Abstract

Tissue engineering aims at developing the necessary technological strategies for replacement or regeneration tissues. However, the number of cells required is much greater than the number obtained from a cell source. Expanding the cells' number in cell culture for a long period is required until the necessary amount of cells is obtained. While in culture, cells often go unwanted differentiation. Little attention has been given to the use of proteolytic enzymes in cell passage. Review the importance of extracellular matrix and surface proteins for cell behavior and the possible mechanisms of cellular changes that may occur due to the use of proteolytic enzymes is an essential issue. Possible alternative to replace these enzymes in cell passage has also been developed.

## Abstract (lay abstract)

Tissue engineering aims at developing strategies for tissue replacement. However, the number of cells required is much greater than the number obtained from a cell source. Expanding the cells' number in cell culture for a long period is required until the necessary amount of cells is obtained but, cells often go unwanted differentiation. Proteolytic enzymes are commonly used in culture and it might lead to such cellular changes. This article aims at reviewing the possible mechanisms of cellular changes that may occur due to the use of proteolytic enzymes and possible alternatives to replace the use of those enzymes.

Tissue engineering aims at developing the necessary technological strategies for replacement or regeneration of organs and tissues, absent or compromised by injury or disease [1].

Since tissue engineering requires a cell source for the construction of organs or tissues, it is crucial that the best way to prevent immunological incompatibility should be found, and this may be obtained using autologous cells. However, the number of cells required for the construction of an organ or tissue is much greater than the number of cells obtained from an autologous donor cell source. In this way, expanding the number of cells in cell culture for a long period is required until the necessary amount of cells is obtained.

Nevertheless, maintaining the cell characteristics throughout the expansion process of cell number in culture is a challenge as cell processing, cell expansion and cell purification protocols have not been established yet [2].

While in culture, cells often have their gene expression altered and the expressed proteins are modified, so the cells undergo unwanted differentiation. In addition, the presence of chromosomal aberrations and decreased cell viability have been described [3,4]. A variety of studies have been carried out in order to understand and control changes in cell culture, and certain assumptions have been raised. First, fetal bovine serum and culture media: the presence of fetal bovine serum in cell culture medium is questionable since there is a lack of characterization of these substances (growth factors), and the amount of such substances as well. Furthermore, the ideal culture media should contain standard nutrients in accordance with the cell type and necessity [5]. Second, the *in vitro* cell culture environment is very different from the *in vivo* environment of the source tissue, for instance: 3D environment, hormones and circulating growth factors [6]. Third, proteolytic enzymes: using these enzymes in the cell passage or tissue digestion promotes the destruction of both the extracellular matrix (ECM) and surface proteins and may, thus, modify signaling [7] and mechanotransduction of signals to the nucleus [8].

This article will focus on the importance of ECM and surface proteins for cell behavior via mechanotransduction and the possible mechanisms of cellular changes that may occur due to the use of proteolytic enzymes, so widely used in cell culture. It also offers some possible alternatives to replace these enzymes as cell sheets and hydrogel 3D culture.

Since it is essential that clinical safety be ensured as these cells may return to patients, it is important to investigate and understand the existing changes in cell culture. We must be certain that the cells do not carry mutations or unwanted differentiations that may cause any pathology in the medium to long-term horizon.

## The influence of ECM & surface protein in cells: mechanotransduction

The means that cell behavior governed by chemical messengers are well established. Surface proteins can function as cellular receptors, which attach to various ligands, such as growth factors, and trigger-specific signaling pathways involved in stem cell differentiation. However, cells are also influenced by adhesive interactions with their surrounding ECM.

A large part of the extracellular space is filled with a complex network of macromolecules, such as proteins and proteoglycans, which form the so-called ECM. Each type of tissue has a specific combination of quality and quantity of these macromolecules, thus each tissue has its own unique ECM composition. Several studies have demonstrated that ECM greatly influences cell development, migration, proliferation, shape and function, i.e., it influences cell behavior and differentiation as well [8–12].

The complex architecture of ECM structural proteins such as fibronectin, collagen and laminin supplies the mechanical properties of the matrix. The environmental changes are transmitted to the cells through the connection between the surface proteins on the cell surface and these ECM proteins [9,10]. Thus, the interactions between mammalian cells and ECM occur via surface proteins. Also, they can function as adhesion molecules, binding to neighboring cells or even to ECM macromolecules [10,11].

In detail, surface proteins are divided in two main classes: Ca^+2^-independent adhesion molecules and Ca^+2^-dependent adhesion molecules. The first group is mainly formed by the immunoglobulin superfamily. NCAM, the best-known molecule, is involved in cell signaling and connection to cytoskeleton [10]. The second group comprises cadherins and integrins. Cadherins are surface proteins responsible for cell–cell adhesion and in most cases they mediate cytoskeletons of adjacent cells interaction [13]. On the other hand, integrins are surface proteins that also connect adjacent cells, but most of them, connect cells to ECM instead of cytoskeleton [14].

Thus, signals transmission from the extracellular environment to the intracellular environment occurs by binding ECM components to a variety of surface proteins. The interplay between the right balance of formation and loss of these complexes as well as the different types of adhesion molecules in each cell forms a key combination for tissue development, maintenance and differentiation [9].

In this way, ECM and surface proteins perform the so-called mechanotransduction, which transmits signals to the cell nucleus and alters gene expression. This occurs because the living cells and the nucleus are so strongly interconnected that a mechanical traction applied to cell surface receptors can immediately change the molecular organization in the cytoplasm and the nucleus. Molecular bonds among integrins, cytoskeleton filaments and nuclear framework may provide a way to mechanically transfer signals through the cells, and a mechanism for producing changes in the cell in response to changes in ECM adhesion [8]. Detailed mechanisms between integrins and ECM interactions can be found in Walters' review [15].

Besides signals mechanotransduction through the cytoskeleton, transcription factors and other signal transducers are connected to the cytoskeleton and the reorganization of the latter, in accordance with extracellular signals, would release transducers and alter gene expression [16].

Therefore, while the ECM was thought to function as structural support for the cells, it is now clear that dynamically reciprocal communication between cells and ECM by surface proteins allows for continual modification of the microenvironment and cell behavior [12]. It is becoming increasingly evident that the cellular microenvironment contributes to the spatial and temporal domains of the signaling complex, directing cellular phenotype.

Studies have shown that phenotype can supersede genotype simply through interactions with the ECM, that is, despite the genetically programmed cell expression, this can be changed by modifying the interaction with the ECM. Nelson and Bissell have shown that the cellular environment is a decisive factor for development of cancer and they gather evidence to show that tissue architecture is as important to tumor growth as DNA mutations. For example, breast cells cancer (carrying DNA mutation in tumor suppressor genes and oncogenes) presenting malignant behavior both in its organizational morphology as in its function was put back in a 3D culture. This 3D culture presented specific components of breast tissue environment. Those cells returned to a normal like organizational morphology and function (producing milk) [9,17]. Thus, the cell can no longer be thought of as a lone entity defined by their genome, but it must be evaluated in the ECM context, soluble growth factors, hormones and other small molecules that regulate organs and functions.

Another example of the transformation that the extracellular environment may generate is found in embryonic cells. The ECM plays an important role in embryonic development, influencing these cells' behavior and differentiation [18]. Embryonic cells are transcriptionally active, showing little chromosome association with histones (more decondensed) and little DNA methylation. During the differentiation process, these cells show increased heterochromatin, the variety of transcription factors and the transcriptional activity decrease, the latter becoming more specific according to each cell type. These complex changes occur by mechanical signals of the environment and are defined by the interaction of the cytoskeleton with adhesion molecules (surface proteins) and by the interaction of the latter with the ECM. Moreover, these changes also occur through biochemical signals into the cell interior of which surface protein participate as surface receptors [18,19].

Furthermore, other studies have shown that the cell shape exerts influence on the cell nucleus, leading to histone acetylation, thereby guiding chromosome condensation and transcriptional activity in specific regions. The ECM directly participates in the cell shape by the connection between both of them [20].

The ECM includes other structural components that have important function and participation in cell behavior. The ECM proteoglycans attract water, which fills the interstitial space of this matrix and sequesters soluble biomolecules such as growth factors and small glycoproteins. These biomolecules in turn bind to integrins and matrix proteins, triggering specific responses. The cells dynamically restructure the microenvironment both for releasing these signaling molecules and cell migration or accommodation. This mechanism works through proteins that cleave the matrix, such as metalloproteinases, and deposition of new matrix components. Regulation is via integrin-mediated signaling pathways [21]. Proteolytic enzymes affect the balance between matrix degradation and matrix deposition during cell passage.

Cell surface glycans are mediators of various receptor–ligand interactions. Recently, it has been demonstrated that several extracellular enzymes, naturally produced, alter glycans structure and, consequently, their ligand-binding properties. This causes a change of transduced signals, reinforcing the importance of receptor–ligand interactions in cell fate and behavior [22].

Generally speaking, cell fate is regulated by the extracellular environment signals, showing when there will be division, differentiation, migration or death. Specific cell receptors interact with proteins, glycans, soluble factors, neighboring cells and guide cell fate, highlighting the importance of conservation of the ECM and cell receptors (surface proteins) [6].

All this context enables identification of the profound changes in stem cell behavior that may be produced using proteolytic enzymes in cell culture because these enzymes not only destroy the ECM but also interfere in surface proteins. However, just a few studies were conduct in order to elucidate the possible changes that those enzymes can trigger in cell behavior.

Huang *et al*. have studied the proteomic changes caused by the use of proteolytic enzymes (such as trypsin) in cell passage. Their findings showed that 36 proteins were differentially expressed in the trypsin-treated cells. Proteins related to the regulation of metabolism, growth, the mitochondria electron transport and cell adhesion showed less expression, while proteins that regulate apoptosis showed more expression [23].

Yang studied morphology, immunophenotype, viability, proliferation, differentiation and the maintenance of membrane proteins of mesenchymal stem cells harvested from glass coverslips with and without the use of proteolytic enzymes. It was conclude that cell detachment without proteolytic enzymes facilitated the maintenance of membrane proteins and preserved mesenchymal stem cells properties as viability, proliferation and differentiation to some extent [6].

These studies lead us to analyze the deep cellular changes that may occur during long-term culture in stem cells. Therefore, alternatives to the use of trypsin are being developed, such as cell culture in cell sheets or hydrogel 3D culture.

## Culture in cell sheets

Cell sheets culture was developed with the aim to promote cell passage without the use of proteolytic enzymes in order to preserve the connection between cells, the ECM and surface proteins. Several studies have been conducted using this new technology aiming to promote tissue regeneration or even produce them in three dimensions without the use of scaffolds.

Yamada together with Okano developed the cell sheets technology, in which the cells and their ECM are collected together, without proteolytic enzymes treatment or any tool for extracting cells. Thus, the surfaces of the well-plates are coated with thermoresponsive polymers, poly(*N*-isopropylacrylamide) or PIPAAm. This polymer changes its cell adhesion property as there is temperature change. At temperature above 32°C, the surfaces become hydrophobic, repelling water in the polymer, making it somewhat thin and thus allowing cell adhesion on the plastic culture. At temperature below 32°C, the surfaces become hydrophilic, drawing water into the polymer, expanding it and inhibiting cell adhesion to the plastic culture, thereby promoting the release of cells joined by their ECM. Several studies with different cell groups have been developed using the cell sheets technology [23–25].

Rat cardiomyocytes, for example, were cultured in cell sheets. Four sheets were layered and exhibited synchronized pulse, suggesting a morphological and electrical connection among the cells. After transplantation, up to week 12, the heartbeat was still present and the cells histologically had heart tissue characteristics with multiple neovascularization [26,27].

Yang reported the use of cell sheets technology to develop functional leaf-shaped tissue. These tissues have been used in various ways such as: corneal impairment, to accelerate healing following esophagus surgery and the trachea epithelium cell sheets transplantation in a Dracon vascular prosthesis after tracheal resection. The group also studied methods to develop 3D tissues using cell sheets only (without scaffolds), reinforcing the advantage of releasing cells without the use of proteolytic enzymes, thus preserving surface proteins and growth factor receptors, ion channels and proteins associated with cell–cell junction [28].

In a attempt to minimize the difficulty in establishing cell–cell contact of primary hepatocytes engrafted with the host tissue in liver therapies, Ohashi used primary hepatocytes cultures in cell sheets. The cells and their matrix were transplanted into the subcutaneous space, and an efficient union between grafted cells and the surrounding cells was obtained. This union persisted for more than 200 days. The newly developed liver tissue also exhibited several features of liver-specific functions. The authors also report the simplicity of the technique as it is minimally invasive and free of immunogenic potential because it does not require the use of scaffolds [29].

The cultured of cell sheets pancreatic islet cells was performed. 76% of those cells were insulin-positive and 19% were glucagon-positive cells. The functionality was preserved for 7 days after transplantation into the subcutaneous space of rats, suggesting the possibility of using cells from cell sheets for diabetes mellitus [30].

A combination of a treated dentin matrix scaffold and dentin follicle cells grown in cell sheets was used in an attempt to promote root regeneration. Cell sheets were preferred for showing a better survival rate of the cells. Histological examination demonstrated the formation of a rich ECM with the presence of collagen I, fibronectin, integrin β1 and alkaline phosphatase. Under the effect of dentin matrix, there was significant expression of *DMP-1* and bone sialoprotein (*BSP*) by cells, indicating odontogenic and osteogenic potential. The dentin matrix could still induce dentin follicle cells to develop a tissue similar to dentin-pulp and cementum-periodontal complex because these cells showed positivity to markers such as nestin, factor VIII and CAP [31].

A comparison between the preservation of the primitive state of bone marrow mesenchymal stem cells and adipose tissue, released by trypsinization or by cell sheets technology was performed. The result of the experiment demonstrated that, after three passages, cells grown in cell sheets preserve both viability and proliferation properties, and differentiation to some extent. It was suggested that keeping the ECM proteins and surface proteins, cell membrane can have a major influence on this result, which does not occur with cell trypsinization [32].

Cell sheets technology may be a new alternative for tissue engineering as a way of enabling pluripotent stem cells expansion without their differentiation for subsequent construction of organs or tissues, according to the needs of each individual.

## Hydrogel 3D culture

The literature related to hydrogel technique is extremely extensive. Here, we opted to provide an overview of its applicability. Hydrogel presents a variety of applicable techniques: It can be used to culture cells in bioreactors [33], as a 3D culture which avoids the use of proteolytic enzymes [34], as a mechanical vehicle to 3D cell/organ printing [35,36] and as a biocompatible material to be implanted *in vivo* as well [37].

Such variety of hydrogel application is due to manageable properties: They can mimic the tissue-specific native cellular microenvironment, for example, the ECM components, molecules such as cytokines and chemokines, and physicochemical conditions (as density, stiffness and degradation rate) [38]. Those properties can be manipulated according to tissue and culture requirements.

Hydrogel is an interesting culture alternative because cells behave more likely to the original sampling tissues when cultured in 3D environment [15]. The unnatural 2D cell culture microenvironment affects intracellular signaling and phenotypic fate [6], since only one side of the cell's membrane is in contact with the ECM and the neighboring cells [37]. For instance, once stem cells are displaced from the niche, they will begin to differentiate into specialized cell types [39].

Cells cultured in 2D do not express certain tissue-specific genes and proteins in comparison to those expressed *in vivo* [40]. Chondrocytes differentiate in 2D culture but, those cells look like their original phenotype when cultured in 3D [41].

A hydrogel 3D culture provides a more natural environment for cell's growth by the additional dimension for external signals, and binding events occurred between integrins on the cell surface and the ECM proteins. However, it is a challenge to promote appropriate oxygen, soluble factors, cells' nutrients transport requirements [42].

Despite the challenges, hydrogel 3D culture and its properties are a good alternative solution for either preservation or manipulation of ECM components as avoiding proteolytic enzymes.

Hydrogel 3D stem cell culture has been developed to better recapitulate many of the ECM/environment signals to guide stem cell behavior, both as *in vitro* models and as delivery vehicles for *in vivo* implantation. The importance of preserving tissue proteins, communication and structure has been gaining attention [43].

Developing hydrogels that mimic natural environment and the ECM of each type of tissue are a huge challenge [6] that has been slowly accomplished. For example, Hanjaya-Putra first demonstrated the capacity of the hydrogel to either support or inhibit *in vitro* vasculogenesis of endothelial cells. Second, they achieved the ability to control degradation cues that regulate vascular tube formation utilizing hyaluronic acid hydrogels [44]. Also, hydrogels have been developed as a repair for cartilage defect, varying in the type of polymer, degradation profile, mechanical properties and loading regimen, source of cells, cell-seeding density, controlled release of growth factors and strategies to cause integration with surrounding tissue [37].

## Conclusion

The knowledge developed in the last two decades maintains that the cell can no longer be thought of as a single entity because its behavior is greatly influenced by the extracellular environment, and by the signals received by the cell. Thus, the preservation of the ECM and surface proteins have gained great importance in the field of cell culture, so that cell behavior destination, differentiation and cell viability can be controlled. The use of proteolytic enzymes needs to be reassessed; cell sheets and hydrogel 3D culture can be interesting alternatives to the use of proteolytic enzymes.

## Future perspective

It has been shown that several genetic alterations are not sufficient to trigger specific phenotypes, and this seems to depend closely on changes in the ECM [8,16,19,45–46]. In this way, the use of scaffold for tissue engineering must be reassessed as they can largely influence cells behavior since scaffold may mimic the extracellular environment. If scaffolds are incompatible with the desired characteristics for a given tissue, they may adversely impact the behavior of this new organ/tissue that was implanted. Custom made or decellularized scaffolds may be an interesting alternative. Furthermore, the use of cells with its preserved matrix could exempt the need of a scaffold.

The nutrients and signaling molecules of a cell medium can interfere in cell signaling and behavior. Standardized cell culture relative the quantity and quality of cell medium's components for each cell type must be developed.

Finally, methods to evaluate cells viability and safety prior to clinical implementation must be evolved.

**Executive summary**

While in culture, cells often go unwanted differentiation. Little attention has been given to the use of proteolytic enzymes in cell passage.
The preservation of the extracellular matrix (ECM) and surface proteins have gained great importance in the field of cell culture.
The use of proteolytic enzymes in cell passage needs to be reassessed.
The cell can no longer be thought of as a single entity because its behavior is greatly influenced by the extracellular environment.
Several studies have demonstrated that the ECM and surface proteins greatly influence cell development, migration, proliferation, shape and function.
Cell sheets can be an interesting alternative to the use of proteolytic enzymes.
Hydrogel 3D culture may be another alternative to avoid the use of proteolytic enzymes.
Tissue-specific hydrogels which mimic natural environment and ECM have been developed.
New technologies which permit the cell passage/culture without using proteolytic enzymes must be developed.
Tissue engineering has created methods to evaluate cells viability and safety prior to clinical implementation.
